# Effects of Chitosan on Intestinal Inflammation in Weaned Pigs Challenged by Enterotoxigenic *Escherichia coli*

**DOI:** 10.1371/journal.pone.0104192

**Published:** 2014-08-04

**Authors:** Dingfu Xiao, Yongfei Wang, Gang Liu, Jianhua He, Wei Qiu, Xionggui Hu, Zemeng Feng, Maoliang Ran, Charles M. Nyachoti, Sung Woo Kim, Zhiru Tang, Yulong Yin

**Affiliations:** 1 College of Animal Science and Technology, Hunan Agricultural University, Changsha, China; 2 Research and Development Center, Twins Group Co., Ltd, Nanchang, Jiangxi, China; 3 Hunan Engineering and Research Center of Animal and Poultry Science and Key Laboratory for Agro-ecological Processes in Subtropical Region, Institute of Subtropical Agriculture, the Chinese Academy of Sciences, Hunan, China; 4 Hunan New Wellful Co., LTD, Changsha, Hunan, China; 5 Hunan Institute of Animal and Veterinary Science, Changsha, China; 6 Department of Animal Science, Faculty of Agricultural and Food Sciences, University of Manitoba, Winnipeg, Manitoba, Canada; 7 Department of Animal Science, North Carolina State University, Raleigh, North Carolina, United States of America; 8 College of Animal Science and Technology, Southwest University, Chongqing, China; Indian Institute of Science, India

## Abstract

The aim of this study was to investigate whether supplementation with chitosan (COS) could reduce diarrhea and to explore how COS alleviates intestinal inflammation in weaned pigs. Thirty pigs (Duroc×Landrace×Yorkshire, initial BW of 5.65±0.27) weaned at age 21 d were challenged with enterotoxigenic Escherichia *coli* during a preliminary trial period, and then divided into three treatment groups. Pigs in individual pens were fed a corn-soybean meal diet, that contained either 0 (control), 50 mg/kg chlortetracycline, or 300 mg/kg COS for 21 days. The post-weaning diarrhea frequency, calprotectin levels and TLR4 protein expression were decreased (*P*<0.05) in both the COS and chlortetracycline groups compared with control. Simultaneously, supplemental COS and chlortetracycline had no effect on the mRNA expression of TNF-α in the jejunal mucosa, or on the concentrations of IL-1β, IL-6 and TNF-α in serum. However, COS supplementation improved (*P*<0.05) the mRNA expression of IL-1β and IL-6 in the jejunal mucosa. The results indicate that supplementation with COS at 300 mg/kg was effective for alleviating intestinal inflammation and enhancing the cell-mediated immune response. As feed additives, chitosan and chlortetracycline may influence different mechanisms for alleviating inflammation in piglets.

## Introduction

Weaning removes young pigs from the passive immune protection they receive from the milk of the sow and increases their susceptibility to enterotoxigenic *E. coli* infection [1]. Early-weaned pigs often exhibit an underdeveloped immune system, digestive disorders and post-weaning diarrhea [2]. Enterotoxigenic *E. coli* not only colonize the small intestine, but can also release enterotoxins to stimulate epithelial cells to secrete fluid into the lumen of the gut to cause diarrhea [3]. Therefore, antibiotics are often added to the diet of weanling piglets to prevent infectious disease and improve growth. However, it has been suggested that the continuous use of antibiotics may contribute to a reservoir of drug-resistant bacteria which may be capable of transferring their resistance to pathogenic bacteria in both animals and humans [4]. In addition, consumers are becoming increasingly concerned about the presence of drug residues in livestock products. As a result, many countries have either banned or are in the process of banning the use of antibiotics in pig diets as a routine method for promoting growth.

The pro-inflammatory cytokines IL-1β, IL-6 and TNF-α play a central role in the cell-mediated immune response, and also participate in the maintenance of tissue integrity [5]. The level of fecal calprotectin is a sensitive and non-invasive marker of active inflammation in the gastrointestinal system [6]. Fecal calprotectin may be increased under various conditions, such as inflammatory bowel disease. TLR4, a key receptor for commensal recognition in gut innate immunity, is over-expressed in inflamed colonocytes and is the subject of therapy (target inhibition) in inflammatory bowel disease [7].

Several reports have described the effects of COS on growth [8], immunity [1], and oxidative stress [9], as well as the antimicrobial [10], hypolipidemic [11], and particularly the anti-inflammatory activities of COS both *in vitro* [12], [13]and *in vivo* [14], [15]. The anti-inflammatory effect of COS *in vitro* has been shown to positively correlate with its molecular weight (MW) and degree of deacetylation (DD) [16]. Chitosan has been shown to have specific immunomodulatory effects: i.e., it can polarize the cytokine balance toward Th1 cytokines, decrease the production of the inflammatory cytokines IL-6 (interleukin-6) and TNF-α, down-regulate CD44 and TLR4 receptor expression, and inhibit T cell proliferation [17]. However, it is still unclear whether dietary supplementation with COS can alleviate inflammatory bowel diseases and how COS affects intestinal inflammation. Our previous studies showed that supplemental COS in weaned piglets decreased the feed conversion ratio and improved the intestinal morphology, intestinal connectivity, and intestinal mucosal immunity [18]. We established an *Escherichia coli* model of post-weaning diarrhea in early-weaned piglets, and used this new model to investigate the effects of COS on intestinal inflammation by daily monitoring of diarrhea and analyzed the effects of COS on inflammatory responses by determining TLR4 and calprotectin protein expression, as well as the concentration and mRNA expression of IL-1β, IL-6 and TNF-α.

## Materials and Methods

### Animals and experimental design

Animals and experimental design were same with the previous reported paper [18]. Thirty 21-day-old piglets (Duroc×Landrace×Yorkshire, initial BW of 5.65±0.27) were challenged with enterotoxigenic *Escherichia coli* during a preliminary trial period. The piglets were then randomly assigned into three groups with 10 piglets in each group. The piglets in the control group (A group) were fed the basal diet without any supplement, those in the chlortetracycline group (B group) were fed the basal diet plus 50 mg/kg chlortetracycline, and those in the COS group (C group) were fed the basal diet plus 300 mg/kg COS. The basal diets were formulated based on NRC requirements (National Research Council, 1998), and their compositions and nutritional levels are listed in Table 1. Each group of piglets was fed their respective diet for 21 days. COS (molecular weight <5,000 Da and degree of deacetylation >90%) was provided by Dalian Chemical and Physical Institute (Chinese Academy of Sciences, city, China) and has a 6-sugar unit of N-acetyl glucosamine with β-(1–4)-linkages. Chlortetracycline was provided by Jinhe Biotechnology Co., Ltd. (city, China).

**Table 1:** Composition and nutrient levels of the basal diet (DM basis) %.

Items	Content
Ingredients	
Corn	58.42
Soybean meal	25.00
Fish meal	5.00
Whey powder	4.00
Cream powder	5.00
Limestone	0.30
CaHPO_4_	1.10
Moldproofant	0.10
Antioxidant	0.02
Vitamin premix1)	0.04
Choline chloride	0.08
Mineral premix2)	0.30
NaCl	0.30
Flavor	0.06
L-Lys HCl	0.23
Met	0.05
Total	100.00
Calculation composition	
DE (MJ/kg)	14.3
CP (%)	19.00
Ca (%)	0.58
AP (%)	0.42
Lys (%)	1.20
Met (%)	0.40
Thr (%)	0.85

1 Provided additional vitamins per kilogram diet: VA 11 000 IU, VD_3_ 1 100 IU, VE 16 IU, VK 1 mg, pantothenate 6 mg, retinoic acid 2 mg, folic acid 0.8 mg, nicotinic acid 10 mg, thiamine 0.6 mg, VB_1_ 0.6 mg, biotin 0.08 mg, VB_12_ 0.03 mg.

2 Provided with additional trace elements per kilogram diet: Zn 165 mg, Fe 165 mg, Mn 33 mg, Cu 16.5 mg, I 297 µg, Se 297 µg.

Piglets were randomly allocated into pens with one pig per pen in a temperature-controlled room, as described by Tang et al. Feed was provided to the piglets three times per day at 8:00, 12:00 and 18:00, and any uneaten food was weighed in the morning of the next day. Feed and water were provided *ad libitum*. The piglets were checked daily for signs of disease and mortality. The animals were weighed individually, and feed intake and feed efficiency were determined for each pen on a weekly basis to monitor the growth of animals fed the different diets. At the end of the 21-day period of feeding with the experimental diets, six piglets per treatment were sacrificed for sampling. The animal protocol was approved by the Animal Care Committee of the Institute of Subtropical Agriculture, the Chinese Academy of Sciences.

### Sampling and sample processing procedures

Six piglets from each treatment group were sacrificed after feed deprivation for 12 h by the injection of 4% sodium pentobarbital solution (40 mg/kg BW) for the collection of tissue samples on day 21 post-weaning. Blood samples were taken from the heart, and were centrifuged at 3000 rpm for 10 minutes, then, the serums were collected and stored at −80°C. The small intestine (SI) was removed and its length was determined; the jejunum was considered to be located at about 50% of the length of the SI. About 3 g of jejunal mucosa was collected immediately, frozen in liquid nitrogen, and stored at −80°C until the extraction of total RNA. Four cm-long segments were excised from the jejunum and fixed in 4% formaldehyde for subsequent morphological and immunohistochemical analysis.

### Diarrhea index

The piglets' stool was observed when they were fed, and those that had thin, soft feces were considered to have diarrhea. Diarrhea index (%) was calculated as 100× number of piglets that had diarrhea/total number of piglets.

### Enzyme-linked immunosorbent assay (ELISA)

Cytokines including IL-1β, IL-6 and TNF-α in serum were measured by an ELISA kit (R & D Systems, Wiesbaden-Nordenstadt, Germany) according to the manufacturer's instructions.

### Immunohistochemistry and relative quantitative real-time PCR

Calprotectin and TLR4 protein expression in the jejunal mucosa were detected by immunohistochemistry. The mRNA expression of IL-1β, IL-6 and TNF-α in the jejunal mucosa were detected by relative quantitative real-time PCR and the results were presented as fold changes using the 2^−ΔΔCT^ method (Table 2). Those methods were reported in the previous paper [18].

**Table 2:** Sequences (5′–3′) of the primers used for the detection of mRNA specific for IL-1β, IL-6, TNF-α and GAPDH.

Gene	Accession number	Primer sequences (5′ to 3′)	Product size (bp)
GAPDH	AF017079	F: GAAGGTCGGAGTGAACGGAT	149
		R: CATGGGTAGAATCATACTGGAACA	
IL-1β	NM_214055.1	F: GAAAGATAACACGCCCACCC	165
		R: TCTGCTTGAGAGGTGCTGATGT	
IL-6	NM_214399.1	F: TCCAGAAAGAGTATGAGAGCA	177
		R: TCTTCATCCACTCGTTCTGT	
TNF-α	NM_214022.1	F: CCACGCTCTTCTGCCTACTGC	168
		R: GCTGTCCCTCGGCTTTGAC	

Notes: F = forward primer; R = reverse primer.

### Statistical Analysis

The data was analyzed by analysis of variance (ANOVA) using the general linear model (GLM) procedure of the statistical analysis system (SAS) programs (9.1). Duncan's multiple range test was applied for comparing the differences among treatments. The difference was considered to be significant at *P*<0.05, and be very significant at *P*<0.01.

## Results

### Diarrhea

**Figure 1:**
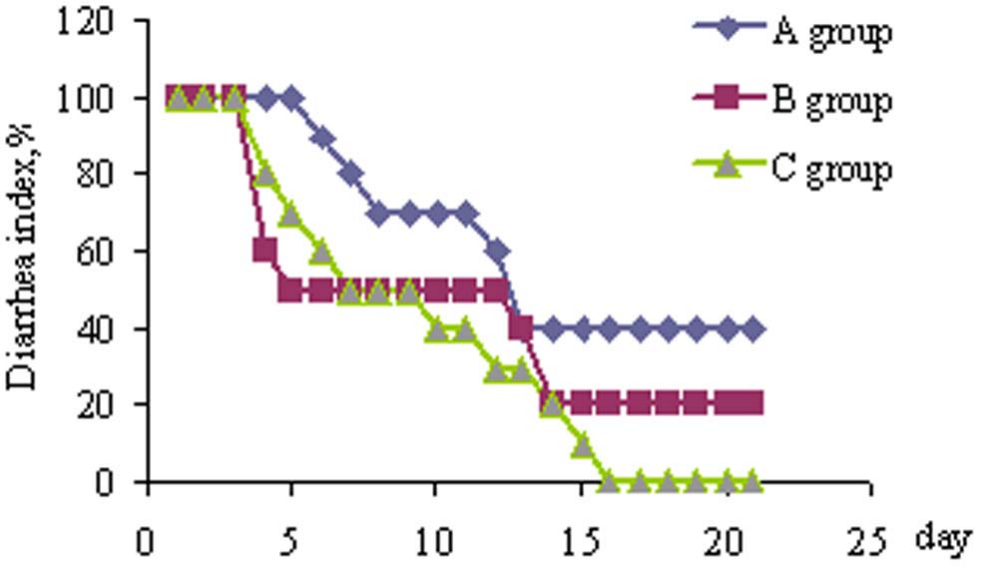
Changes in diarrhea index in piglets. Piglets challenged by enterotoxigenic *Escherichia* coli were fed either a control diet (A group, n = 10), or control diet plus chlortetracycline (B group, n = 10) or control diet plus chitosan (C group, n = 10) for 21 days. Diarrhea index (%) was calculated as 100× number of piglets that had diarrhea/total number of piglets.

As presented in Fig. 1, the diarrhea frequency slowly decreased as the experiment progressed, and four piglets had diarrhea at the end of the feeding trial in the control group. In the chlortetracycline group, five piglets had diarrhea on the fifth day, and from day 17 to the end, two piglets had diarrhea; In the COS group, from day 14 to the end, none of the piglets had diarrhea. The results showed that 300 mg/kg COS had a similar effect (*P*>0.05) on reducing diarrhea as 50 mg/kg chlortetracycline.

### Inflammatory cytokines

The concentrations of IL-1β, IL-6 and TNF-α in serum were not affected by any of the treatments (Table 3).

**Table 3:** Serum IL-1β, IL-6 and TNF-α levels in weaned piglets.

Items	Treatments^1^			SEM	*P*-value
	A group	B group	C group		
IL-1β (ng/L)	58.36	65.14	61.65	1.783	0.3000
IL-6 (ng/L)	57.20	63.35	67.30	1.808	0.133
TNF-α (ng/L)	54.84	60.01	61.34	1.709	0.441
Number of observations	6	6	6		

1 A group means the control group, B group means the chlortetracycline group, C group means the COS group.

### Jejunal mucosal calprotectin and TLR4 protein expression

**Figure 2:**
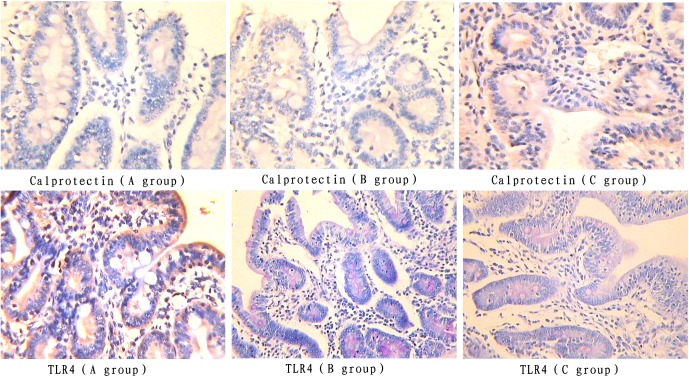
Calprotectin and TLR4 protein expression in jejunal mucosa (immunohistochemical staining, ×400). Piglets challenged by enterotoxigenic *Escherichia* coli were fed either a control diet (A group, n = 6), or control diet plus chlortetracycline (B group, n = 6) or control diet plus chitosan (C group, n = 6) for 21 days and 6 pigs per treatment were killed to excise the jejunum.

The color signals of jejunal mucosal calprotectin and TLR4 protein expression in the COS group were lighter than those in both the control and chlortetracycline groups (Figure 2). The integral optical density of calprotectin and TLR4 protein expression in the COS group was lower (*P*<0.05) than those in both the control and chlortetracycline groups, and there was no difference between the chlortetracycline group and the control group (Table 4).

**Table 4:** Integral optical density of Calprotectin and TLR4 protein expression in jejunal mucosa.

Items	Treatments^1^			SEM	*P*-value
	A group	B group	C group		
Integral optical density of Calprotectin protein expression	389.68^a^	327.54^b^	338.65^b^	17.49	0.0285
Integral optical density of TLR4 protein expression	250.51^a^	202.30^b^	212.94^b^	12.52	0.0397
Number of observations	6	6	6		

a, b Means with different superscripts in the same row differ (*P*<0.05).

1 A group means the control group, B group means the chlortetracycline group, C group means the COS group.

### Jejunal mucosal IL-1β, IL-6 and TNF-α mRNA expression

**Figure 3:**
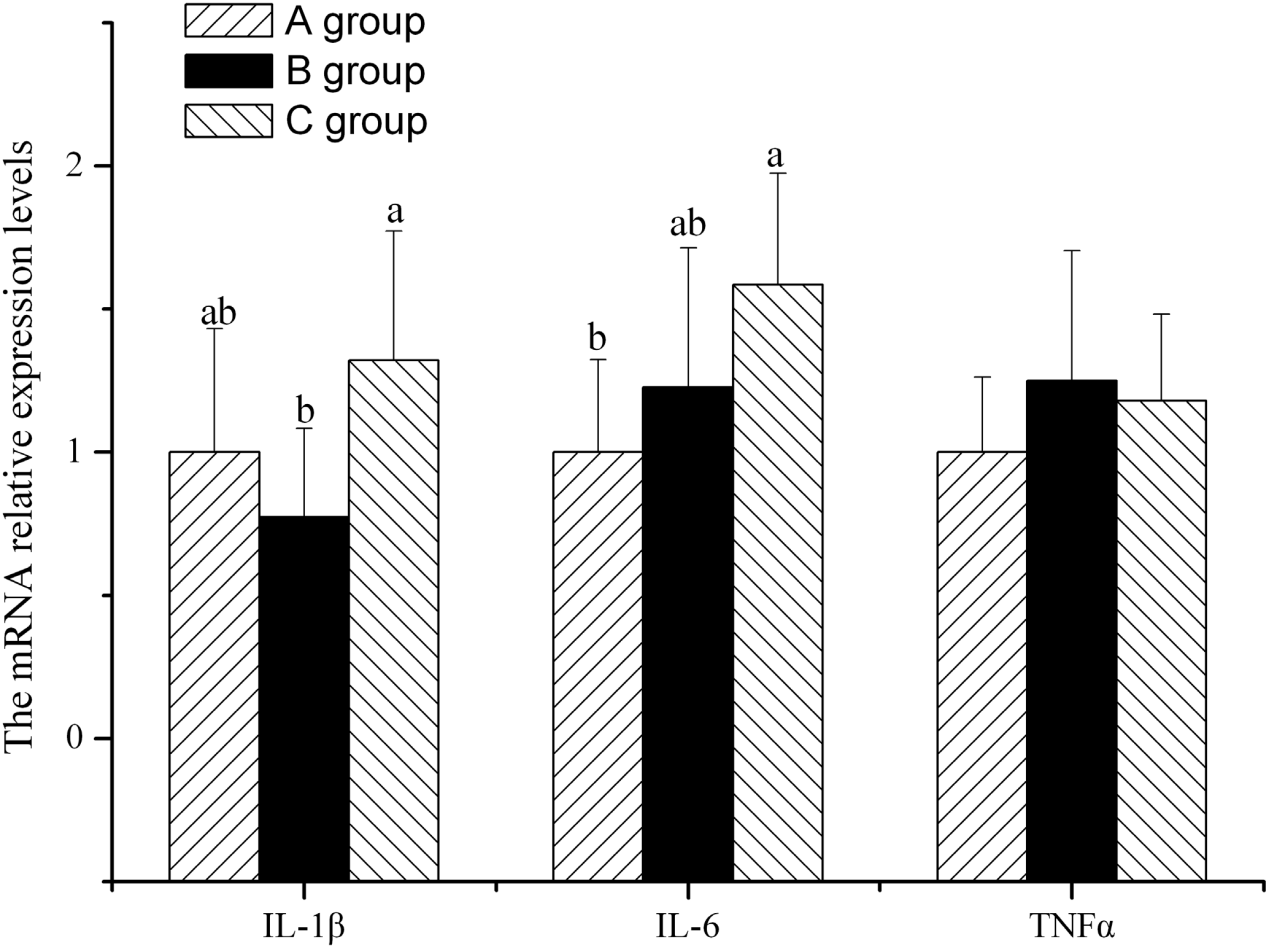
The relative expression of IL-1β, IL-6 and TNF-α mRNA in jejunal mucosa. Piglets challenged by enterotoxigenic *Escherichia* coli were fed either a control diet (A group, n = 6), or control diet plus chlortetracycline (B group, n = 6) or control diet plus chitosan (C group, n = 6) for 21 days and 6 pigs per treatment were killed to excise the jejunum.

There was no difference in the relative expression level of jejunal mucosal TNF-α mRNA among the three groups (Figure 3). The relative expression level of jejunal mucosal IL-6 mRNA in the COS group was higher (*P*<0.05) than that in the control group. There was no difference between the chlortetracycline group and the COS group (Figure 3). The relative expression level of jejunal mucosal IL-1β mRNA in the COS group was higher (*P*<0.05) than that in the chlortetracycline group. There was no difference between the chlortetracycline group and the COS group (Figure 3).

## Discussion

The present study established an excellent piglet diarrhea model for intestinal disorder and demonstrated a clear difference between the effects of COS or chlortetracycline on intestinal inflammation. Chito-oligosaccharide reduced the incidence of diarrhea, but the growth performance of *E. coli*-challenged pigs supplemented with 160 mg of chito-oligosaccharide was not better than that of unsupplemented pigs challenged with *E. coli* K88. Dietary supplementation with COS at 100 and 200 mg/kg enhanced growth performance by increasing apparent digestibility, decreasing the incidence of diarrhea, and improving the small intestine morphology [19].

In essence, the danger model proposes that endogenous host-derived molecules from damaged cells and tissues activate the immune system to cause a systemic inflammatory response. Activation of the corresponding receptors in turn results in the production of pro-inflammatory and tissue-injurious mediators [20]. Toll-like receptors (TLRs) are an important class of pattern recognition receptors (PRRs) in innate immunity, and play a critical role in pathogen recognition and host defense [21]. TLR4 activation and cytokine production by intestinal epithelial cells (IECs) can induce the recruitment and activation of inflammatory cells, and prolonged or dysregulated pro-inflammatory cytokine production may lead to tissue damage and epithelial barrier dysfunction [22]. TLR4 plays a major role in controlling inflammation through the inhibition of mitogen-activated protein kinase (p38 and c-Jun N-terminal kinase) and NF-κB signaling pathways [23]. TLR4 activation has been shown to be involved in the pathogenesis of acute tissue injury and the induction of a systemic inflammatory state [24]. An important biological consequence of TLR signalling is the production of chemoattractants, which leads to the recruitment of inflammatory cells to the site of exposure [25]. Therefore, this piglet diarrhea model was useful for studying TLR4-mediated inflammatory responses in the intestine. Our earlier work showed that COS supplementation decreased the expression of *TLR4* mRNA, and our current work extends these findings by demonstrating that COS supplementation decreased TLR4 protein expression, which indicates that COS supplementation can efficiently activate an inflammatory immune response, thus reducing intestinal infection.

Calprotectin is a cytosolic protein in the S-100 protein group; its levels increase under conditions such as inflammation, infection, and malignancy. It has immunomodulatory, antimicrobial and antiproliferative action and is predominantly found in neutrophils, monocytes and macrophages, as well as (to a lesser extent) in T and B lymphocytes [26]. Fecal calprotectin is a promising marker of neutrophilic intestinal inflammation [27], and correlates well with the severity of inflammation, as judged by both endoscopic and histological scoring systems [28]. It has been consistently shown to be elevated in patients with known irritable bowel disease (IBD), and has an excellent negative predictive value in ruling out IBD in under-diagnosed symptomatic patients. The degree of inflammation has been shown to be strongly associated with calprotectin-positive cells in the stomach [29]. *E. coli* elicited a significant increase in the calprotectin level, which was confirmed by immunofluorescence and immunohistochemistry, and the calprotectin level is an important indicator of inflammatory bowel disease [5]. Calprotectin is an abundant neutrophil protein that is released during inflammation. Since calprotectin correlates well with the degree of inflammation, the results of the current study demonstrated that supplementation with COS and chlortetracycline efficiently decreased calprotectin protein expression, and therefore inhibited inflammation and decreased diarrhea in piglets.

Proinflammatory cytokines emanating from the immune system can have profound effects on the neuroendocrine system, either by gaining direct access to the central nervous system or by triggering the synthesis of cytokines by cells in the central nervous system [30]. Inflammatory activation (IL-1β or TNF-α) of astrocytes results in the transient production of key inflammatory mediators including IL-6, cell surface adhesion molecules, and various leukocyte chemoattractants [31]. Lower levels of IL-6 help to regulate the recruitment and activity of inflammatory cells and limit inflammatory damage. As an innate immune receptor, TLR4 activates NF-κB through recruitment of the adaptor proteins myeloid differentiation primary response gene 88 (MyD88) and Toll/interleukin 1 receptor domain-containing adaptor protein inducing IFN-β (TRIF), which leads to the subsequent induction of NF-κB signalling genes, such as TNF-α, IL-1 and IL-6 [32]. The complex network of cytokines regulates the immune response in the host to prevent susceptibility to disease and enhance resistance to infections. Logically, up-regulation of the protein expression of TLR4 should indicate that the TLR4 signaling pathway is inhibited, and its downstream cytokines including IL-1β, IL-6 and TNF-α should decrease. However, IL-1β and IL-6 mRNA expression improved in that study. Moreover, that study supported our present study in that COS supplementation promoted IL-1β and IL-6 expression, while COS did not affect either TNF-α mRNA expression in the jejunal mucosa, or the levels of IL-1β, IL-6 and TNF-α in serum. We can speculate on possible reasons for these results: In response to peripheral challenge with enterotoxigenic *E. coli*, comprehensive inflammation may have been elicited in weaned piglets; a variety of cells in the immune system may have secreted high concentrations of proinflammatory cytokines. COS supplementation enhances the cell-mediated immune response by modulating the production of cytokines, but some time is needed before TLR4 can activate its downstream signaling pathway. Therefore, IL-1β and IL-6 mRNA expression in intestinal mucosa were still quite high when the piglets were slaughtered. This result is consistent with a previous report that supplementation with COS and chlortetracycline have similar effects in reducing intestinal inflammation, but different effects on intestinal mucosal barrier function [18].

In summary, we can propose a mechanism by which COS can inhibit diarrhea: after chitosan adheres to the intestinal mucosa, its amine is recognized by the immune system. Immune response pathways are activated, and the gut-associated lymphoid immune system is stimulated to produce lymphokines and inflammatory mediators, secrete cytokines IL-1, IL-6, etc., reduce calprotectin and TLR4 protein expression, and enhance the cell-mediated immune response. Therefore, COS helps to prevent inflammatory intestinal disorders, including weaning-associated intestinal inflammation. Furthermore, our previous paper reported that diets supplemented with COS or chlortetracycline could improve intestinal mucosal morphology and occludin protein expression [18]. Thus, the digestion and absorption of nutrients in the intestine are improved in piglets. These may be contributing factors for chitosan to decrease diarrhea.

In conclusion, we have demonstrated that supplementation with COS and chlortetracycline can decrease the occurrence of diarrhea and alleviate intestinal inflammation by up-regulating TLR4 and calprotectin protein expression in weaned piglets challenged with enterotoxigenic *Escherichia* coli. In addition, COS can enhance the cell-mediated immune response by modulating the production of inflammatory cytokines.

## References

[1] Yin YL, Tang ZR, Sun ZH, Liu ZQ, Li TJ, et al (2008) Effect of Galacto-mannan-oligosaccharides or chitosan supplementation on cytoimmunity and humoral immunity in early-weaned piglets. Asian-Aust J Anim Sci 21: 723–731.

[2] Fairbrother JM, Nadeau E, Gyles CL (2005) Escherichia coli in postweaning diarrhea in pigs: an update on bacterial types, pathogenesis, and prevention strategies. Anim Health Res Rev 6: 17–39. 16164007 10.1079/ahr2005105

[3] Liu P, Piao XS, Thacker PA, Zeng ZK, Li PF, et al (2010) Chito-oligosaccharide reduces diarrhea incidence and attenuates the immune response of weaned pigs challenged with Escherichia coli K88. J Anim Sci 88: 3871–3879. 20656977 10.2527/jas.2009-2771

[4] van der Fels-Klerx HJ, Puister-Jansen LF, van Asselt ED, Burgers SL (2011) Farm factors associated with the use of antibiotics in pig production. J Anim Sci 89: 1922–1929. 21606448 10.2527/jas.2010-3046

[5] Splichal I, Fagerhol MK, Trebichavsky I, Splichalova A, Schulze J (2005) The effect of intestinal colonization of germ-free pigs with Escherichia coli on calprotectin levels in plasma, intestinal and bronchoalveolar lavages. Immunobiology 209: 681–687. 15804046 10.1016/j.imbio.2004.09.009

[6] von Roon AC, Karamountzos L, Purkayastha S, Reese GE, Darzi AW, et al (2007) Diagnostic precision of fecal calprotectin for inflammatory bowel disease and colorectal malignancy. Am J Gastroenterol 102: 803–813. 17324124 10.1111/j.1572-0241.2007.01126.x

[7] Heimesaat MM, Fischer A, Jahn HK, Niebergall J, Freudenberg M, et al (2007) Exacerbation of murine ileitis by Toll-like receptor 4 mediated sensing of lipopolysaccharide from commensal Escherichia coli. Gut 56: 941–948. 17255219 10.1136/gut.2006.104497 PMC1994376

[8] Tang ZR, Yin YL, Nyachoti CM, Huang RL, Li TJ, et al (2005) Effect of dietary supplementation of chitosan and galacto-mannan-oligosaccharide on serum parameters and the insulin-like growth factor-I mRNA expression in early-weaned piglets. Domest Anim Endocrinol 28: 430–441. 15826777 10.1016/j.domaniend.2005.02.003

[9] Anandan R, Ganesan B, Obulesu T, Mathew S, Kumar RS, et al (2012) Dietary chitosan supplementation attenuates isoprenaline-induced oxidative stress in rat myocardium. Int J Biol Macromol 51: 783–787. 22829055 10.1016/j.ijbiomac.2012.07.016

[10] Rabea EI, Badawy ME, Stevens CV, Smagghe G, Steurbaut W (2003) Chitosan as antimicrobial agent: applications and mode of action. Biomacromolecules 4: 1457–1465. 14606868 10.1021/bm034130m

[11] Kobayashi S, Terashima Y, Itoh H (2002) Effects of dietary chitosan on fat deposition and lipase activity in digesta in broiler chickens. Br Poult Sci 43: 270–273. 12047092 10.1080/00071660120121490

[12] Yoon HJ, Moon ME, Park HS, Im SY, Kim YH (2007) Chitosan oligosaccharide (COS) inhibits LPS-induced inflammatory effects in RAW 264.7 macrophage cells. Biochem Biophys Res Commun 358: 954–959. 17512902 10.1016/j.bbrc.2007.05.042

[13] Pangestuti R, Bak SS, Kim SK (2011) Attenuation of pro-inflammatory mediators in LPS-stimulated BV2 microglia by chitooligosaccharides via the MAPK signaling pathway. Int J Biol Macromol 49: 599–606. 21704648 10.1016/j.ijbiomac.2011.06.014

[14] Fernandes JC, Spindola H, de Sousa V, Santos-Silva A, Pintado ME, et al (2010) Anti-inflammatory activity of chitooligosaccharides in vivo. Mar Drugs 8: 1763–1768. 20631868 10.3390/md8061763 PMC2901823

[15] Qiao Y, Bai XF, Du YG (2011) Chitosan oligosaccharides protect mice from LPS challenge by attenuation of inflammation and oxidative stress. Int Immunopharmacol 11: 121–127. 21059391 10.1016/j.intimp.2010.10.016

[16] Lee SH, Senevirathne M, Ahn CB, Kim SK, Je JY (2009) Factors affecting anti-inflammatory effect of chitooligosaccharides in lipopolysaccharides-induced RAW264.7 macrophage cells. Bioorg Med Chem Lett 19: 6655–6658. 19846296 10.1016/j.bmcl.2009.10.007

[17] Chen CL, Wang YM, Liu CF, Wang JY (2008) The effect of water-soluble chitosan on macrophage activation and the attenuation of mite allergen-induced airway inflammation. Biomaterials 29: 2173–2182. 18279951 10.1016/j.biomaterials.2008.01.023

[18] Xiao D, Tang Z, Yin Y, Zhang B, Hu X, et al (2013) Effects of dietary administering chitosan on growth performance, jejunal morphology, jejunal mucosal sIgA, occluding, claudin-1 and TLR4 expression in weaned piglets challenged by enterotoxigenic Escherichia coli. Int Immunopharmacol 17: 670–676. 24007779 10.1016/j.intimp.2013.07.023

[19] Liu P, Piao XS, Kim SW, Wang L, Shen YB, et al (2008) Effects of chito-oligosaccharide supplementation on the growth performance, nutrient digestibility, intestinal morphology, and fecal shedding of Escherichia coli and Lactobacillus in weaning pigs. J Anim Sci 86: 2609–2618. 18502883 10.2527/jas.2007-0668

[20] Reino DC, Palange D, Feketeova E, Bonitz RP, Xu da Z, et al (2012) Activation of toll-like receptor 4 is necessary for trauma hemorrhagic shock-induced gut injury and polymorphonuclear neutrophil priming. Shock 38: 107–114. 22575992 10.1097/SHK.0b013e318257123a PMC3378823

[21] Shimazu T, Villena J, Tohno M, Fujie H, Hosoya S, et al (2012) Immunobiotic Lactobacillus jensenii elicits anti-inflammatory activity in porcine intestinal epithelial cells by modulating negative regulators of the Toll-like receptor signaling pathway. Infect Immun 80: 276–288. 22083706 10.1128/IAI.05729-11 PMC3255675

[22] Hormannsperger G, Haller D (2010) Molecular crosstalk of probiotic bacteria with the intestinal immune system: clinical relevance in the context of inflammatory bowel disease. Int J Med Microbiol 300: 63–73. 19828372 10.1016/j.ijmm.2009.08.006

[23] Byun EB, Sung NY, Byun EH, Song DS, Kim JK, et al (2013) The procyanidin trimer C1 inhibits LPS-induced MAPK and NF-kappaB signaling through TLR4 in macrophages. Int Immunopharmacol 15: 450–456. 23261363 10.1016/j.intimp.2012.11.021

[24] Mollen KP, Anand RJ, Tsung A, Prince JM, Levy RM, et al (2006) Emerging paradigm: toll-like receptor 4-sentinel for the detection of tissue damage. Shock 26: 430–437. 17047512 10.1097/01.shk.0000228797.41044.08

[25] Sahlander K, Larsson K, Palmberg L (2012) Daily exposure to dust alters innate immunity. PLoS One 7: e31646. 22355383 10.1371/journal.pone.0031646 PMC3280315

[26] Beser OF, Sancak S, Erkan T, Kutlu T, Cokugras H, et al (2014) Can Fecal Calprotectin Level Be Used as a Markers of Inflammation in the Diagnosis and Follow-Up of Cow's Milk Protein Allergy? Allergy Asthma Immunol Res 6: 33–38. 24404391 10.4168/aair.2014.6.1.33 PMC3881398

[27] Costa F, Mumolo MG, Bellini M, Romano MR, Ceccarelli L, et al (2003) Role of faecal calprotectin as non-invasive marker of intestinal inflammation. Dig Liver Dis 35: 642–647. 14563186 10.1016/s1590-8658(03)00381-5

[28] Konikoff MR, Denson LA (2006) Role of fecal calprotectin as a biomarker of intestinal inflammation in inflammatory bowel disease. Inflamm Bowel Dis 12: 524–534. 16775498 10.1097/00054725-200606000-00013

[29] Choi HK, Lee YH, Park JP, Min K, Park H (2013) Inflammatory responses in the muscle coat of stomach and small bowel in the postoperative ileus model of guinea pig. Yonsei Med J 54: 1336–1341. 24142636 10.3349/ymj.2013.54.6.1336 PMC3809856

[30] Liu Y, Chen F, Li Q, Odle J, Lin X, et al (2013) Fish oil alleviates activation of the hypothalamic-pituitary-adrenal axis associated with inhibition of TLR4 and NOD signaling pathways in weaned piglets after a lipopolysaccharide challenge. J Nutr 143: 1799–1807. 24005609 10.3945/jn.113.179960

[31] van Kralingen C, Kho DT, Costa J, Angel CE, Graham ES (2013) Exposure to Inflammatory Cytokines IL-1beta and TNFalpha Induces Compromise and Death of Astrocytes; Implications for Chronic Neuroinflammation. PLoS One 8: e84269. 24367648 10.1371/journal.pone.0084269 PMC3868583

[32] Dou W, Zhang J, Sun A, Zhang E, Ding L, et al (2013) Protective effect of naringenin against experimental colitis via suppression of Toll-like receptor 4/NF-kappaB signalling. Br J Nutr 110: 599–608. 23506745 10.1017/S0007114512005594 PMC3726555

